# Thirteen quick tips for bacteriophage genome annotation

**DOI:** 10.1371/journal.pcbi.1014887

**Published:** 2026-10-26

**Authors:** Bishoy Maher Zaki, George Bouras, Andrew M. Kropinski, Alice R. Wattam, Robert A. Edwards, Ramy K. Aziz

**Affiliations:** 1 Bioscience Research Laboratories, MARC for Medical Services and Scientific Research, Giza, Egypt; 2 School of Medicine, College of Health, Adelaide University, Adelaide, South Australia, Australia; 3 The Department of Surgery-Otolaryngology Head and Neck Surgery, Central Adelaide Local Health Network, Adelaide, South Australia, Australia; 4 Department of Pathobiology, Ontario Veterinary College, University of Guelph, Guelph, Ontario, Canada; 5 Biocomplexity Institute, University of Virginia, Charlottesville, Virginia, United States of America; 6 Bacterial and Viral Bioinformatics Resource Center (BV-BRC), Computing, Environment and Life Sciences, Argonne National Laboratory, Lemont, Illinois, United States of America; 7 Flinders Accelerator for Microbiome Exploration, Flinders University, Bedford Park, South Australia, Australia; 8 Department of Microbiology and Immunology, Faculty of Pharmacy, Cairo University, Cairo, Egypt; Montreal, CANADA

Bacteriophages (phages) are ubiquitous, highly diverse viruses that contribute ~25% of the total nucleotides on Earth [1]. Although they exceed bacteria in abundance and diversity, their genomes remain underrepresented in public databases, and knowledge about their functional potential is lagging. There are at least two reasons for this: First, more bacteria are sequenced for their perceived human value(s), either because they infect humans, are used in food production, or make biotechnologically useful products. Second, because bacteriophages lack core metabolic pathways and marker genes, their annotation is always challenging, and the portion of genes with completely unknown functions (dark matter [2]) is generally higher than that in any other domain of life. The awareness of these challenges sparked some initiatives to increase the representation of environmental phages and improve their annotation (e.g., the SEA-PHAGES program [3]), but even such initiatives introduce bias on their own, leading to higher representation of phages infecting a handful of bacterial hosts [4].

While phages share certain genomic characteristics with their prokaryotic hosts, some differences require tailored annotation approaches for their genomes, including differences in open reading frame (ORF) structures, intergenic spaces (or lack thereof), and median coding sequence (CDS) sizes [5,6]. With many tools being specifically developed for phage genome annotation, “manual” verification remains necessary to ensure accuracy and biological relevance [7].

Phage genome annotation becomes even more confusing when incorrect, misleading, or ambiguous data infiltrate genomic databases, driving the propagation of annotation errors. Therefore, there is a growing need for guidelines to minimize errors and maintain genomic database integrity. In this article, we focus on the most common type of phage, tailed ones, grouped under the class of *Caudoviricetes* [8], and provide ***13 quick tips*** that ensure good quality annotation.

## Foundational principle: Annotate with future applications in mind

Like with many endeavors in life, it is important to “begin with the end in mind!” (Quoted from the bestseller “The 7 Habits of Highly Effective People” [9]). Annotating bacteriophage genomes to expand our knowledge of phage biodiversity has different approaches than doing so for an anthropocentric purpose, such as exploiting the bacteriophage for biocontrol, therapy, or as a source of bioactive proteins (lysins, depolymerases, or molecular biology enzymes).

Once this principle is established, the following **13 quick tips** stand as pillars supporting effective bacteriophage genome annotation.

### Tip 1. Learn about your system before you start: Study the basics of phage biology and genomics

It sounds intuitive: understanding the fundamentals of genomics and phage biology is essential for accurate phage genome analysis. Bioinformatics expertise and experience with cellular organisms are great competencies that still need a boost of phage biology basics. The basics include understanding the functions of different phage protein families and modules, when they are expressed, and how they interact to perform their roles. It is equally important to understand how protein structures relate to their function (see **tip #12**). Specific structural features, such as transmembrane domains and the existence of signature conserved motifs, can greatly assist in the accurate annotation of some gene products (e.g., phage lysins and holins). Turner *et al*. [10], Shen and Millard [11], and SEA-PHAGES [12] provide useful resources that can help you develop a deep understanding of the basics of phage biology and genomics.

### Tip 2. Quality check raw data and pursue optimal coverage depth

Quality control of raw sequencing data and accurate phage genome assembly are indispensable steps for reliable annotation [7]. As expected, low sequencing depth and low-quality reads may lead to incorrect assembly; but excessively high sequencing depth may also negatively affect assembly. In high-depth data sets, sequencing errors, rare contaminants (e.g., a second closely related phage or prophage sharing homologous regions with the target phage), and microdiversity (including single nucleotide polymorphisms) may become represented by sufficient number of reads to affect the assembly, especially in repetitive regions in phage genomes. These highly represented regions may increase the assembly graph complexity, which may lead to fragmented or incorrect assemblies including chimeric contigs.

Because excessively high-coverage depth is common with phage genomes, owing to their relatively small sizes, subsampling to 25–100× coverage depth is recommended for phages sequenced with Illumina short reads [10,13]. However, the optimal depth range remains less clearly established for long-read sequencing, and it depends on the applied workflow [14]. Regardless of the sequencing technology, a successful complete genome assembly depends on the assembly algorithm, as the same data set can generate a complete genome with an assembler and a fragmented one with another [13–16]. If initial assembly is fragmented or suspected to be erroneous, apply different algorithms and compare their outputs based on completeness, number, and length of contig(s), assembly-graph structure, read-mapping depth and coverage, and alignment with closely related genomes [10,11,17].

In addition, the coverage depth patterns across an assembled phage genome and orientation of read starts can be used to predict phage termini and DNA packaging mechanisms with tools, such as PhageTerm [18,19]. However, termini prediction is impeded by transposase-based library preparation (e.g., Nextera XT), because this technology modifies the natural physical ends of the phage genome [10].

It is possible to separate the target phage DNA from its host’s (or from other sequences belonging to other phages) based on the coverage depth homogeneity of the assembled contigs: reads from the same phage/biological source tend to have even coverage, unlike those from the host or contaminating viruses. Normally, your target contig(s) should have significantly higher coverage than the background sequences. You can also explore possible assemblies and verify purity by metagenomic binning.

Generally, short-read sequencing is more reliable in phage genome sequencing because it provides higher accuracy per base than long-read platforms. In contrast, long-read sequencing can better resolve genome structure and terminal repeats, but it may still require extensive polishing [10]. Although hybrid co-assembly appears to be a promising solution for generating complete genome assemblies, it may fail with genomes containing complex structures, such as direct terminal repeats (DTRs). To overcome this problem, Elek *et al*. developed a workflow named HYPPA (**HY**brid and **P**oly-polish **P**hage **A**ssembly), in which they perform long-read assembly first, to resolve the genome structure, then proofread by sequential long- and short-read polishing with multiple tools. HYPPA outperformed traditional hybrid co-assembly and generated complete genomes of DTR-containing T7-like phages [14].

### Tip 3. Inspect and organize your files

No less important than preprocessing and quality checking raw data is organizing your intermediate and output files. Since multiple rounds of optimization are often needed to achieve the best results throughout the annotation pipeline, clear file naming and tracking are required. We suggest creating a folder for each step (e.g., trimming, assembly, automated annotation, manual verification), with files named and dated according to settings and main outcomes. In file naming, we recommend the use of underscores, not spaces or hyphens (e.g., “Assembly_SPAdes_OneContig_20250319”), so you can easily use them in your scripts. Note that here we use “big-endian” (ISO 8601) date format, which avoids global ambiguity and sorts seamlessly by date. Spreadsheets can also help organize comparisons between different tools for manual verification of the functional assignment of predicted CDSs (Also check **tips #7 and 9**).

### Tip 4. Use automated annotation tools with healthy skepticism

One needs to be aware that major automated annotation pipelines were developed to annotate bacterial genomes not those of phages. This has progressively changed with the introduction of PhANOTATE [20], Phage Commander [21], Pharokka [22], PhaGAA [23], PhageScope [24], Empathi [25], and the BV-BRC phage pipeline [26], to name a few. Many are freely available with user-friendly web interfaces that significantly facilitate the annotation process and help avoid common errors, such as over-annotation [10,27]. Other tools are available through command-line access and are thus powerful with processing files in batches and can also be included in analysis pipelines. However, automated annotation is not error-free; therefore, manual verification is essential to ensure accuracy [7].

### Tip 5. Use phage-aware tools and comparative approaches for accurate gene calling

Because phage genomes are highly compact, with coding sequences that often overlap or are separated by short non-coding intergenic regions, it is essential to carefully evaluate non-coding gaps (over 150 bp in length) and overlaps during annotation. Typically, the coding capacity of phage genomes reaches 90%–95%; if it appears substantially lower than this, your phage genome may utilize an alternative genetic code [28]. Additionally, short CDSs (< 100 bp) may be overlooked by prokaryotic genome annotators, as the statistics supporting the interpretation of these regions as functional genes are often indistinguishable from those of non-coding sequences, and the signal is too weak to distinguish them from random genomic background [29]. While phage-specific gene-calling tools (like PhANOTATE [20]) exist, they produce slightly different results. To improve annotation reliability, comparing results from multiple annotation tools is valuable.

Another approach to validate gene calling is to consider the most closely related RefSeq phage genomes or those recognized by the International Committee on Taxonomy of Viruses (ICTV) as references. You can initially identify closely related phage genomes among the best BLASTn hits with high query coverage and nucleotide identity to your candidate genome. However, there are no universal BLASTn cutoffs to define reference phages for annotation. For screening, we recommend hits with coverage ≥ 70% and identity ≥ 80%, although the ideal approach is to plot the distribution of coverage and identity values, then to look for hits that statistically stand out. A more systematic approach to select closely related reference phages is to use whole-genome intergenomic similarity tools, such as VIRIDIC, which applies ICTV cutoffs for clustering genera (≥70%) and species (≥ 95%) [30].

This comparative approach to gene calling helps determine which potential CDSs should be retained, as well as their correct start sites, as start sites are often conserved among homologs. Additionally, preferred start sites are typically preceded by an upstream Shine-Dalgarno sequence (ribosome-binding site, RBS) at a spacing of 3–7 bases [10].

### Tip 6. Check for embedded CDSs, programmed ribosomal frameshifting, introns, and inteins

Automated annotation tools usually overlook embedded CDSs and other phage-specific features, such as programmed ribosomal frameshifting (PRF), introns and inteins [31]. These unusual genetic elements or features necessitate ‘manual’ verification during phage genome annotation. For instance, some types of spanins are encoded by embedded ORFs. In such a case, the o-spanin-encoding gene is entirely embedded within the i-spanin-encoding gene [32].

PRF is a translational feature that has been reported in some phages, like coliphages T7 and lambda [33]. In PRF, a specific sequence motif, known as ‘slippery sequence,’ facilitates ribosomal slippage (commonly a –1 frameshift) into an alternative reading frame, allowing translation to proceed and produce an additional longer protein. When closely related phages are available in the databases, BLASTx analysis can identify potential frameshifts [10]. The conservation of the frameshift motif across closely related phage genomes and the presence of well-mapped reads to this region support PRF prediction, which would otherwise be regarded as sequencing or assembly errors.

Introns and inteins have been documented in phage genomes, with varying frequency among phage groups. For example, they are frequent among some crAss-like phages [34]. To detect these elements, we recommend PRFect [35] along with BLASTx analysis of the predicted coding sequences [10]. If the resulting hits are homologous to more than one protein that matches different regions in the query, this suggests embedded introns or inteins. Additionally, differences between the ORF length and the expected protein size may indicate the presence of introns or inteins [36].

### Tip 7. Avoid and rename ambiguous functional assignments

Clear, meaningful names for predicted gene products are essential to prevent confusion and enhance effective communication. First, remember that you annotate functions not proteins, i.e., one protein may have multiple functions (e.g., fusion proteins). Second, avoid using non-descriptive labels, such as arbitrary numbers, codes, or generic terms like “gp##,” as these often refer to different encoded functions across various phages, leading to ambiguity [11]. Third, be consistent in gene product naming, follow ICTV guidelines for naming, and check trusted databases, such as UniProtKB [37] and ViralZone [38] for the appropriate ontology/ controlled vocabulary [10,39].

### Tip 8. Annotate locally; think globally

When naming your gene products, think globally. Even if annotating one or a few phage genomes, the function you will assign to one protein will be propagated to all its homologs. This concept has led to a general paradigm shift in genome annotation by adopting subsystems annotation or annotation across gene families, rather than genome walking [40]. Thus, it is highly recommended not to include a phage’s name or any information related to its geography in the functional role assigned to one of its proteins. Global thinking also includes taking metagenomics analyses into consideration: a protein’s functional role can be assigned to its closest homolog in a metagenomic data set. The more descriptive and specific the function is, the better it will help other investigators make sense of the sequences they discover in the most diverse ecosystems.

### Tip 9. Evaluate functional annotations critically to avoid error propagation

Many phage genes are unique: they have no homologs in any database. Consequently, it is quite normal for most annotated CDSs to be assigned as hypothetical proteins. Some annotation enthusiasts mistakenly believe that a high-quality annotation is one with *more* functional assignments and *fewer* hypotheticals, even in the absence of sufficient supporting evidence. Informally, this can be described as: “A hopeful lie is better than a sad truth.” Such practices contribute to muddling the databases with misleading annotations and, even more dangerously, to the propagation of annotation errors.

Therefore, it is better to be conservative when assigning functions based solely on BLASTp results [10]. More is not always better! For example, ensure that your protein’s length aligns with the average length of similar proteins with database hits. E-values < 1 × 10^-5^ are generally considered relevant, especially if the alignment covers most or all of the protein amino acid sequence. However, thresholds are always arbitrary, and nothing supersedes visual inspection of the alignments and the statistical distribution of E-values. Additional evidence, such as conserved domains, is required to assign functions with high confidence. If you are not confident, do not assign a function. Labeling a protein as hypothetical is usually easier to modify than assigning an incorrect function to that protein.

### Tip 10. Context matters: Analyze gene synteny and chromosomal context for more relevant functional assignments

Phage genomes are often organized into functional modules, where genes with related functions are clustered, frequently forming operons [41]. This arrangement facilitates coordinated expression of functionally linked genes. Therefore, gene synteny (the conserved order of genes) is an important factor in solving the puzzle of phage genome annotation. You can check gene synteny by comparing your phage genome to other RefSeq or ICTV-recommended genomes, using tools such as progressiveMauve [42], clinker [43], or LoVis4u [44], and by screening the PHROGS database [45] for homologs of the neighboring genes of your predicted CDS homologs. You can also use Phynteny, an annotation tool that uses gene synteny to predict functional assignments [46].

### Tip 11. Use the right tools to infer safety and therapeutic potential of your phage

The suitability of phages for therapeutic applications requires a comprehensive assessment of their genomes to ensure safety and efficacy. Phages for therapeutic use should not harbor genes encoding virulence-associated toxins (i.e., encoded by converting phages and pathogenic in eukaryotic hosts) or antimicrobial resistance proteins [11].

Additionally, current protocols require therapeutic phages to be strictly lytic, with no ability to establish lysogeny—mainly through integrating into the bacterial genome [11]. However, in some cases, you may observe your phage acting strictly lytic on an overlay plate, forming clear plaques, while some lysogeny markers (such as genes encoding integrases, repressors, antirepressor proteins, excisionases, or replicative transposases) are still identified in its genome. The failure of a phage to lysogenize its bacterial host is not solely determined by the phage genome; many temperate phages require host-specific factors to establish lysogeny, e.g., bacterial attachment sites (*attB*), whose absence would favor a lytic life cycle [47]. Conversely, the lysogenic markers could be non-functional remnants inherited from temperate ancestors [48]. Over time, these genes may accumulate mutations that render them non-functional while the phage continues to replicate exclusively through the lytic cycle. For that purpose, look for evidence of gene decay by checking for premature stop codons, frameshift insertion-deletions (indels), loss of conserved functional domains or fragmentation compared to intact homologs. Comparing your phage genome with closely related ones helps avoid misinterpretation due to potential sequencing or assembly errors.

Furthermore, the presence of diversity-generating retroelements (DGRs) in phage genomes is a concern, as these elements can induce mutations that may unpredictably alter phage-host interactions [49,50]. Several bioinformatics tools and databases have been developed to screen for these genetic markers [50–55].

### Tip 12. Integrate structure prediction into your phage genome annotation workflow

Key phage proteins often have characteristic structural topologies that are associated with their functions. For instance, spanins and holins, which are involved in host lysis and lack enzymatic domains, are characterized by specific topological patterns of transmembrane domains, signal peptides, coiled-coil motifs, and beta sheets [32,56], while depolymerases are also characterized by their own folds [57–61]. These proteins are highly variable among phages; however, analyzing their structural features can provide evidence for their function. Tools that can predict simple structural features based on the amino acid sequence include InterProScan [62], Phobius [63], and TMHMM [64]. More recent AI-based tools, such as DeepTMHMM [65] and SignalP 6.0 [66], can identify features in more evolutionarily distant proteins.

The life of phage annotators has remarkably improved since the introduction of the AI-based AlphaFold series [67]. Integrating structure prediction tools into phage genome annotation workflows, such as Phold [68], has enhanced functional predictions, leading to more reliable annotations, especially for phage structural proteins, e.g., head, neck, baseplate, and tail proteins [68].

### Tip 13. Cross-verify your annotations with multiple tools

A final tip, which applies to all computational prediction tools, is to embrace and master the habit of exploiting redundancy then cross-verifying with multiple tools. In other words, consider all outputs but never rely on a single tool.

The reliability of functional annotations can be enhanced by the integration of multiple bioinformatics tools, each of which provides a unique dimension, especially those based on different algorithms. For example, InterProScan provides functional analysis by classifying proteins into families and predicting domains and important sites, such as transmembrane domains, signal peptides, and coiled-coil structural motifs [62,69]. HHpred analyzes remote protein homology and structure, by comparing profile HMMs of query sequences against those of known proteins in four databases [70], of which we recommend PDB [71], Pfam-A [72], NCBI Conserved Domains [73], and PHROGs [45]. UniProt BLAST identifies regions of similarity between a query protein sequence and sequences in the UniProt and Swiss-Prot databases [74]. Foldseek [75] quickly predicts protein structural information then identifies regions of structural homology between the query and protein structures in various databases, including AlphaFold Database [76], PDB and BFVD [77].

## Conclusion

As the field of phage genomics grows rapidly, more researchers contribute to depositing their phage sequences in public databases. Therefore, setting standards for genome annotation guarantees accountability and reliability. Staying up to date with the latest tools and guidelines is essential for providing comprehensive and accurate annotations. We offer these 13 tips to the phage community to help avoid errors, stop their propagation, and deposit reliable phage genomic data. We finally summarize those tips in three different ways, that can serve as user reference. Table 1 lists all tools and databases discussed within the 13 tips, including their main applications and how to access them. An “annotator checklist” is provided (Box 1) as a cheat sheet for novice and seasoned annotators alike. Finally, a companion interactive HTML file (Supplementary S1 File) serves as a more detailed guide for the 13 tips.

**Table 1:** Summary of bioinformatics tools and databases discussed in the 13 tips.

Tool or database	Main use in phage genome annotation	System/access	Relevant tip(s)
PhageTerm	Prediction of phage genome termini and DNA-packaging mechanisms from sequencing-read coverage and start-position patterns	Command line; available through Galaxy	Tip 2
HYPPA	Hybrid and sequential polishing workflow for complete phage genome assembly, including genomes with direct terminal repeats	Command line	Tip 2
BV-BRC Metagenomic Binning	Isolation of viral and bacterial genome bins from metagenomic reads or assembled contigs	Web server	Tip 2
PHANOTATE	Phage-specific prediction of protein-coding genes in compact genomes	Command line	Tips 4 and 5
Phage Commander	Comparison and integration of predictions from multiple gene-calling programs	Standalone software	Tip 4
Pharokka	Automated, phage-specific genome annotation	Command line	Tip 4
PhaGAA	Automated phage genome annotation	Web server	Tip 4
PhageScope	Automated phage genome analysis, annotation, and visualization	Web server	Tip 4
Empathi	Functional annotation of phage proteins through embedding-based hierarchical assignment	Standalone software	Tip 4
BV-BRC phage pipeline	Automated phage genome annotation	Web server	Tip 4
NCBI RefSeq	Source of curated reference phage genomes for gene calling, start-site evaluation, and comparative annotation	Database	Tips 5 and 10
ICTV taxonomy resources	Identification of officially classified phages and taxonomically relevant reference genomes	Web resource	Tips 5 and 10
BLASTn	Identification of closely related phages through nucleotide similarity, query coverage, and sequence identity	Web server	Tip 5
VIRIDIC	Calculation of pairwise whole-genome intergenomic similarity for species- and genus-level clustering	Web server	Tip 5
ShineFind	Identification of candidate Shine–Dalgarno sequences upstream of coding-sequence start sites	Web server, available through Galaxy	Tip 5
BLASTx	Translated nucleotide comparison for investigating interrupted genes, introns, inteins, and potential frameshifts	Web server	Tip 6
PRFect	Prediction of programmed ribosomal frameshifts	Command line	Tip 6
UniProtKB	Database of curated protein names, functions, and standardized nomenclature	Database	Tips 7 and 13
ViralZone	Virus-specific protein information, functional descriptions, and controlled terminology	Web resource	Tip 7
BLASTp	Preliminary identification of protein homologs and evaluation of potential functional assignments.	Web server	Tip 9
progressiveMauve	Whole-genome alignment and evaluation of conserved gene order	Standalone software	Tip 10
clinker	Comparison and visualization of homologous gene clusters and conserved gene synteny	Web server	Tip 10
LoVis4u	Comparison and visualization of multiple genomic loci, sequence similarity, conserved gene order, GC content, GC skew, and sequencing-coverage profiles	Command line	Tip 10
PHROGs	Phage-specific protein-family database for remote-homology searches and functional-context analysis	Database	Tips 10 and 13
Phynteny	Gene-synteny-based prediction of functional assignments in bacteriophage genomes	Command line	Tip 10
MyDGR	Identification and characterization of diversity-generating retroelements	Web server	Tip 11
PhageLeads	Computational screening of phage genomes for therapeutic suitability	Web server	Tip 11
PhaBOX	Identification and characterization of phage sequences, including phage lifestyle and genomic features	Web server	Tip 11
ResFinderFG	Detection of antimicrobial-resistance genes represented in a functional-metagenomics database	Database and web server	Tip 11
ResFinder	Detection of acquired antimicrobial-resistance genes in nucleotide sequences	Web server	Tip 11
VirulenceFinder	Detection of known virulence-associated genes in nucleotide sequences	Web server	Tip 11
Sphae	Automated assessment of phage genomes and prediction of potential phage-therapy candidates	Command-line toolkit	Tip 11
DePP	Prediction of phage depolymerase proteins	Command line and web server	Tip 12
DepoRanker	Prediction of candidate *Klebsiella* phage depolymerases	Web server	Tip 12
DepoScope	Prediction of phage depolymerase proteins	Command line	Tip 12
DposFinder	Prediction of phage depolymerase proteins and their associated host capsular serotypes	Command line	Tip 12
PhageDPO	Prediction of phage depolymerase proteins	Web server	Tip 12
Phobius	Combined HMM-based prediction of signal peptides and transmembrane topology from protein sequences	Web server	Tip 12
TMHMM	HMM-based prediction of α-helical transmembrane helices and membrane topology from protein sequences	Web server	Tip 12
DeepTMHMM	Deep-learning, protein-language-model-based prediction of α-helical and β-barrel transmembrane proteins and their topology	Web server	Tip 12
SignalP 6.0	Protein-language-model-based prediction of signal peptides and their cleavage sites, covering the five major signal-peptide types	Web server	Tip 12
AlphaFold	AI-based prediction of three-dimensional protein structures	Web server	Tip 12
Phold	Structure-informed functional annotation of phage proteins and genomes	Command line	Tip 12
InterProScan	Classification of proteins into families and prediction of domains, conserved sites, and selected sequence features	Web server and database	Tips 12 and 13
HHpred	Detection of remote protein homology through profile hidden Markov model comparisons	Web server	Tip 13
Protein Data Bank (PDB)	Source of experimentally determined protein structures for homology and structural comparisons	Database	Tip 13
Pfam-A	Curated profile hidden Markov models for protein-family and domain identification	Database	Tip 13
NCBI Conserved Domain Database	Identification of conserved protein domains and functional sites	Database	Tip 13
UniProt BLAST	Protein similarity searches against UniProtKB and its manually reviewed Swiss-Prot section	Web server	Tip 13
Foldseek	Rapid comparison of predicted or experimentally determined protein structures	Web server or command line	Tip 13
AlphaFold Protein Structure Database	Repository of predicted protein structures for structural-homology searches	Database	Tip 13
BFVD	Repository of predicted viral protein structures for structural comparison	Database	Tip 13

Thirteen is a prime number with unusually strong cultural associations; however, the exact number of tips does not matter much: you can add your own tips and create your own phage annotation workflow!

**Box 1: Phage Annotator’s Checklist**

**Before you wrap up the annotation of your phage genome, ensure that:**

- Your raw data have been quality checked (QC’d) and preprocessed/filtered accordingly (Tip 2)
- Your assembly’s coverage depth has been inspected and tested for evenness (Tip 2)
- Your files are visually inspected and screened for major errors (Tip 3)
- Your intermediate and output files are organized in folders to simplify archiving and subsequent validation (Tip 3)
- At least two (the more the better) automated annotation tools have been run and their output appropriately saved so that annotations can be compared (Tip 4)
- Various annotations have been tabulated to allow the choice of the most appropriate (not necessarily the most popular) one (Tip 4)
- A phage-aware gene caller (not a default prokaryotic one) has been used for ORF/gene finding (Tip 5)
- Genomic features that are more common in phage genomes have been analyzed (Tip 6):
  - Embedded CDSs
  - Programmed ribosomal frameshifts
  - Introns
  - Inteins
- Ambiguous functional assignments (e.g., gp## or locus nnn) have been renamed (Tip 7)
- Functional assignments/annotations have been revised for consistency and compatibility with cross-taxon propagation (Tip 8)
- Phage names have been removed from protein annotations (Tip 8)
- Proteins with insufficient evidence are renamed to hypothetical proteins (Tip 9)
- A tool for synteny/chromosomal context analysis has been used for improving the annotations, notably of hypothetical proteins (Tip 10)
- The suitability for phage for therapy has been checked (Tip 11)
  - Lack (or decay) of lysogeny markers
  - Lack of bacterial toxins
  - Lack of horizontally transferred antimicrobial resistance genes
- DGRs have been screened and evaluated (Tip 11)
- A tool for structure prediction has been used for improving the annotation of hypothetical or generic structural proteins (Tip 12)
- A final check of gene products, with a focus on hypothetical proteins, has been performed by tools for motif finding etc. (Tip 13)

## Use of Generative AI

The authors did not use generative AI in drafting or revising the manuscript’s text. On the other hand, an interactive HTML file has been generated by ChatGPT (GPT‑5.6 Sol; OpenAI) to convert the 13 tips into an interactive workflow with hyperlinked resources.

## Supporting information

S1 File A companion HTML file includes the foundational principle and the 13 tips, with decision maps or workflows for each of them. (HTML)

## Abbreviations

CDS: coding sequence
DGRs: diversity-generating retroelements
DTRs: direct terminal repeats
HYPPA: HYbrid and Poly-polish Phage Assembly
ICTV: International Committee on Taxonomy of Viruses
ORF: open reading frame
PRF: programmed ribosomal frameshifting.
