# Supplementary material for: Thirteen quick tips for bacteriophage genome annotation
Source: PLoS Comput Biol. 2026 Oct 26;22(10):e1014887. doi: 10.1371/journal.pcbi.1014887 (PMC13704836; doi:10.1371/journal.pcbi.1014887)
Supplement: Supplementary file 1 [file pcbi.1014887.s001.html]

Interactive Tips for Bacteriophage Genome Annotation


# Quick Tips for Bacteriophage Genome Annotation

Explore one foundational principle and 13 practical annotation tips

Bishoy Maher Zaki · George Bouras · Andrew M. Kropinski · Alice R. Wattam · Robert A. Edwards · Ramy K. Aziz

Explore the tips
All tips at a glance
Print / Save as PDF

Manuscript-derived content is condensed for interactive use. “Workflow checkpoints” are synthesis prompts rather than verbatim manuscript text. Tools and resources reflect the final manuscript and its Table 1.

**Foundation + 13 tips**select a tip


ALL TIPS AT A GLANCE

Browse the complete collection, then select any card to open its detailed guidance.

Interactive visualization based on the final manuscript *Thirteen quick tips for bacteriophage genome annotation*. Generated by ChatGPT (GPT‑5.6 Sol; OpenAI) to convert the foundational principle and 13 tips into interactive decision maps and workflows with supporting resource descriptions.
